# Trauma Care Provision in Malaysia: Challenges, Strengths, and Strategic Priorities for System Reform

**DOI:** 10.7759/cureus.84107

**Published:** 2025-05-14

**Authors:** Daniel I Koshy, David Koshy, Kevin Vinod Joseph

**Affiliations:** 1 Trauma and Orthopaedics, Royal London Hospital, London, GBR; 2 Orthopaedics, University Hospital Plymouth, Plymouth, GBR; 3 Orthopaedics, St John of God (SJOG) Midland Public Hospital, Perth, AUS; 4 Internal Medicine, Addenbrooke's Hospital, Cambridge University Hospitals NHS Foundation Trust, Cambridge, GBR

**Keywords:** emergency medicine services, health policy, malaysia, rehabilitation, rural healthcare, trauma, trauma registry

## Abstract

Trauma remains a leading cause of morbidity and mortality in Malaysia, primarily due to road traffic accidents, falls, and blunt force injuries. Despite the country’s universal healthcare coverage and the establishment of major trauma centres in urban areas, significant disparities in trauma outcomes persist, particularly between urban and rural populations. This narrative review evaluates the current state of trauma care in Malaysia, identifying key strengths, such as accessible public healthcare and structured training programs, while also highlighting critical gaps in emergency medical services (EMS), the absence of a national trauma registry, and limited access to post-trauma rehabilitation. Strategic recommendations include strengthening pre-hospital emergency care, developing a centralised trauma registry, and expanding rehabilitation services, particularly in underserved rural regions. Addressing these challenges through coordinated policy reform, investment in infrastructure, and cross-sectoral collaboration is essential for reducing trauma-related mortality and ensuring equitable access to high-quality care across Malaysia.

## Introduction and background

Trauma is a primary global public health concern and one of the leading causes of death and disability, particularly in low- and middle-income countries (LMICs), where more than 90% of injury-related deaths occur. Malaysia, as an upper-middle-income country, faces a disproportionate burden of trauma, with road traffic accidents (RTAs), falls, and blunt force injuries being the primary contributors [1]. RTAs alone have shown a consistent upward trend in incidence and severity over the past decade, significantly impacting emergency services and the broader healthcare system [2]. In 2012, national statistics showed that RTAs accounted for over 6,000 fatalities annually, with many more sustaining serious injuries requiring hospitalisation or long-term care [2].

Although Malaysia has made considerable progress in expanding access to healthcare through its universal health coverage and has developed trauma care infrastructure in major urban centres, systemic challenges persist. These include stark geographic disparities in access to trauma services between urban and rural areas, fragmented pre-hospital emergency medical services [3], and a lack of standardised national trauma data collection through a central registry [4]. In addition, post-injury rehabilitation services remain underdeveloped and inequitably distributed, limiting long-term recovery and functional outcomes for many trauma survivors [5].

Given these challenges, there is an urgent need to critically examine the trauma care system in Malaysia to identify strengths, weaknesses, and strategic opportunities for reform. This narrative review aims to assess the current trauma care landscape in Malaysia, focusing on three key domains: pre-hospital emergency services, the development of a national trauma registry, and access to post-trauma rehabilitation. This paper proposes evidence-informed recommendations to strengthen trauma care delivery and improve equity and outcomes across the Malaysian health system by synthesising available literature and contextual insights.

## Review

Strengths of trauma provision in Malaysia

Universal Healthcare System

Malaysia’s universal healthcare system is a foundational strength that underpins trauma care access nationwide. The public sector provides heavily subsidised care through government hospitals and clinics, ensuring that essential services, including emergency trauma treatment, are available to all citizens, regardless of socioeconomic status. This model plays a crucial role in trauma scenarios, where time-sensitive interventions, such as surgery, blood transfusion, and critical care, are often unaffordable in private settings.

The Ministry of Health (MOH) budget covers trauma care in public hospitals, which allocates funding to over 140 government hospitals nationwide. A study by Sethi et al. (2002) comparing trauma care between tertiary and secondary facilities in Malaysia found that although resource availability varied, financial accessibility remained consistent due to public subsidies [6].

Moreover, universal coverage reduces financial barriers during the critical “golden hour,” when rapid access to resuscitation and surgery significantly affects survival rates. Countries with similar public financing models, such as Thailand and Sri Lanka, have demonstrated comparable improvements in trauma outcomes due to early access to care without out-of-pocket delays [3,5].

Establishment of Specialised Trauma Centres

Malaysia has invested significantly in developing major trauma centres, especially urban tertiary referral hospitals. These include institutions such as Hospital Kuala Lumpur, Hospital Sungai Buloh, and University Malaya Medical Centre, which serve as key trauma hubs for their regions. These centres are equipped with operating theatres, intensive care units (ICUs), diagnostic imaging (including CT and MRI), and multidisciplinary trauma teams including surgeons, emergency physicians, and rehabilitation staff [1,7].

The availability of specialised trauma centres is positively correlated with improved patient outcomes. Studies have shown that trauma patients treated in designated centres have lower mortality rates, shorter ICU stays, and improved functional recovery [1,7]. Malaysia’s trauma centre model follows international best practices by integrating emergency surgery, critical care, and early rehabilitation under one roof.

However, it is worth noting that these centres are concentrated in urban locations, limiting rural access, a concern discussed further later in this article.

Implementation of Structured Training Programs

Another major strength is the systematic implementation of trauma training for healthcare professionals. The Advanced Trauma Life Support (ATLS) and Prehospital Trauma Life Support (PHTLS) programs are widely adopted in Malaysian hospitals and emergency medical services (EMS) [8]. These programs, developed by the American College of Surgeons and the National Association of EMTS, offer standardised, evidence-based protocols for trauma assessment, airway management, haemorrhage control, and shock resuscitation.

Another major strength is the systematic implementation of trauma training for healthcare professionals. The Advanced Trauma Life Support (ATLS) and Prehospital Trauma Life Support (PHTLS) programs are widely adopted in Malaysian hospitals and emergency medical services (EMS) [8]. These programs, developed by the American College of Surgeons and the National Association of EMTs, offer standardised, evidence-based protocols for trauma assessment, airway management, haemorrhage control, and shock resuscitation. While these protocols are highly effective and globally recognised, it is important to consider their applicability within the local context. For instance, in the United States, where these programs originated, there is a high incidence of penetrating trauma, which heavily informs the training content. According to the Malaysian Ministry of Health's Trauma Registry Report for 2011-2012, blunt trauma accounted for 92.4% of all trauma cases, with motorcycle crashes being a significant contributor. Similarly, the National Trauma Database's first-year report indicated that 83.9% of trauma cases were due to blunt injuries, predominantly from road traffic accidents involving motorcyclists and pillion riders [9]. Therefore, aligning trauma education and training with the epidemiology of local injury patterns is crucial for optimal outcomes.

Several Malaysian studies have shown that ATLS-trained providers demonstrate improved triage accuracy, decision-making, and procedural competence [7,8]. Moreover, the Ministry of Health has mandated regular simulation-based training for emergency staff, ensuring that knowledge and skills remain current. This emphasis on continuing professional development is in line with WHO recommendations for strengthening trauma systems in LMICs.

Weaknesses in trauma provision in Malaysia

Geographic Disparities in Access to Trauma Care

One of the most pressing challenges facing Malaysia’s trauma care system is the marked disparity in access between urban and rural populations. While major cities, such as Kuala Lumpur, Penang, and Johor Bahru, are equipped with tertiary trauma centres offering 24/7 surgical and critical care services, many rural and remote communities, particularly in East Malaysia (Sabah and Sarawak), and parts of the interior peninsula lack even basic trauma care infrastructure. This urban-rural divide leads to significant delays in accessing trauma care, as patients in rural areas must often be transferred to urban hospitals for treatment.

Although some helicopter emergency medical services (HEMS) exist in Malaysia, primarily through the Malaysian Fire and Rescue Department (Bomba) and the Ministry of Health, they are limited in number and not widely available for routine trauma evacuation, especially in remote areas [10,11]. As a result, patients from these regions often face delays exceeding several hours before reaching definitive care, mainly due to a combination of poor road infrastructure, limited ground ambulance availability, and long distances to tertiary centres. These delays have substantial clinical consequences: trauma care delivered beyond the "golden hour" is associated with increased mortality, a higher risk of secondary complications (e.g., sepsis, multiple organ failure), and long-term disability [12,13]. According to the WHO Global Status Report on Road Safety, LMICs like Malaysia also experience elevated pre-hospital death rates, often due to delayed or inadequate interventions [12].

Fragmented Pre-Hospital Emergency Medical Services (EMS)

Malaysia's EMS system, while functional in urban areas, remains fragmented and underdeveloped nationally. Multiple agencies, including the Ministry of Health, St. John Ambulance, Malaysian Red Crescent, and private providers, operate independently, leading to overlapping jurisdictions, inconsistent protocols, and duplication of services. Moreover, there is no centralised dispatch system across states, resulting in inefficiencies and prolonged response times.

In urban areas like Klang Valley, response times average 10-15 minutes. In contrast, in rural Sabah and Sarawak, ambulances may take over 40 minutes to arrive if they are available at all, which leaves access to EMS services limited [13,14]. Compounding this issue is a chronic shortage of trained paramedics. Many ambulances are staffed by drivers with only basic first aid knowledge rather than certified emergency medical technicians (EMTs).

Chew and Chan (2011) emphasised the lack of standardised training and credentialing among EMS personnel in Malaysia, noting wide variation in trauma assessment, airway management, and fluid resuscitation practices [15]. This inconsistency reduces the efficacy of pre-hospital stabilisation and complicates triage and handover to hospital teams.

Furthermore, ambulance equipment is not uniformly regulated. Essential trauma equipment, such as cervical collars, automated external defibrillators (AEDs), spinal boards, and suction devices, may be absent in lower-tier ambulances. This directly contradicts WHO recommendations, which stress that EMS systems must be capable of advanced life support for trauma victims to reduce mortality [3]. This is concerning, as the quality of care provided in the pre-hospital phase can significantly impact patient outcomes.

Analysing the first-year report of the National Trauma Database (NTrD) in Malaysia found that approximately 30% of trauma deaths were considered preventable. These preventable deaths were primarily attributed to delays in treatment and insufficient resources at the point of care [9]. This highlights significant gaps in the trauma care system, particularly in pre-hospital care and timely access to definitive treatment.

Limited Access to Post-trauma Rehabilitation

Another critical weakness in Malaysia’s trauma system is the inadequate availability and distribution of post-traumatic rehabilitation services. Rehabilitation is an essential component of trauma recovery, especially for patients with spinal cord injuries, traumatic brain injuries (TBI), amputations, and polytrauma. However, many government hospitals lack integrated rehab units, particularly in secondary and district facilities.

According to the National Institute for Health and Care Excellence (NICE), rehabilitation should begin early in the acute phase of trauma treatment and continue post-discharge [16]. In Malaysia, however, continuity of care is often disrupted. Only a few urban hospitals, such as University Malaya Medical Centre and Hospital Rehabilitasi Cheras, offer comprehensive services, including physiotherapy, occupational therapy, and neurorehabilitation.

A review by Ong et al. (2022) found that many trauma patients, especially in rural regions, are discharged home without any follow-up rehabilitation, due to workforce shortages, cost barriers, or geographic inaccessibility [17]. This leads to avoidable long-term disability, dependence, and poor quality of life. Rural patients are particularly disadvantaged due to the absence of community-based rehabilitation centres and the unavailability of transportation to distant urban facilities.

Innovative solutions, such as tele-rehabilitation and mobile rehabilitation units, have been piloted in other LMICs with positive outcomes but have yet to be widely implemented in Malaysia [18]. Policymakers must prioritise investment in rehabilitation infrastructure and workforce development, especially in underserved regions.

Priority areas for improving trauma provision in Malaysia

Enhancing Pre-hospital Care

Pre-hospital care is a crucial determinant of trauma outcomes, particularly within the "golden hour", when timely and appropriate interventions significantly reduce mortality and morbidity [3]. In Malaysia, pre-hospital care remains underdeveloped and inconsistent, especially in rural and remote regions such as Sabah and Sarawak. Fragmented emergency medical services (EMS), insufficient ambulance fleets, inadequate equipment, and a lack of trained personnel severely limit the effectiveness of trauma response [14].

To address these issues, Malaysia should prioritise the establishment of a centralised, standardised EMS framework under the Ministry of Health [17]. Such a system should include expansion of ground and air ambulance services with region-specific deployment strategies, integration of EMS dispatch with geographic information systems (GIS) for optimised response times, standardised training for paramedics and emergency responders using internationally recognised protocols like Prehospital Trauma Life Support (PHTLS) and International Trauma Life Support (ITLS) [8], community first responder training programs in remote areas to provide basic life-saving interventions prior to EMS arrival.

Countries like Thailand and India have successfully implemented scalable EMS systems tailored to urban and rural contexts [19]. Malaysia could draw on these models for adaptation. Additionally, establishing performance metrics, such as response time and survival-to-discharge rates, would enable continuous quality improvement [15]. A study by Razzak et al. (2019) highlights that countries that adopted national EMS protocols saw a 15-30% reduction in pre-hospital trauma mortality [20].

Developing a National Trauma Registry

Malaysia currently lacks a national trauma registry, hindering its ability to systematically collect, analyse, and utilise trauma data for clinical and policy decision-making. The absence of reliable, standardised data limits efforts to identify high-risk populations, monitor outcomes, or assess resource utilisation [21].

A national trauma registry should be developed with the following components comprehensive inclusion criteria covering all moderate to severe injuries treated in emergency departments and hospitals, standardised data collection protocols based on 10th revision of the International Classification of Diseases (ICD-10) or Abbreviated Injury Scale (AIS) coding systems, integration with EMS, emergency departments, surgical units, and rehabilitation services, real-time digital platforms allowing secure and efficient data entry across institutions [9].

Examples such as the U.S. National Trauma Data Bank (NTDB) and the UK’s Trauma Audit and Research Network (TARN) demonstrate the value of trauma registries in improving outcomes. In Southeast Asia, countries like Thailand and Vietnam have initiated similar registries with support from the WHO [20]. Trauma registries in LMICs have been associated with enhanced policymaking, more efficient resource allocation, and reductions in trauma mortality by up to 20% [21]

The establishment of Malaysia’s registry will require significant investments in IT infrastructure, personnel training, data governance, and institutional coordination. The involvement of stakeholders from the Ministry of Health, academic institutions, private healthcare providers, and professional bodies will be crucial. International partnerships may provide technical assistance and funding.

Expanding Post-trauma Rehabilitation Services

Rehabilitation is an essential component of trauma care that facilitates functional recovery and social reintegration. Despite its importance, post-trauma rehabilitation in Malaysia remains underdeveloped, especially in non-urban regions. Challenges include a shortage of rehabilitation centres, uneven distribution of trained professionals, and limited integration of rehabilitation into the trauma care continuum [22].

Improving rehabilitation access requires a multifaceted approach. The following steps can be taken to achieve this: increasing the number and geographic distribution of rehabilitation centres, especially in underserved states, expanding the workforce through scholarships, training programs, and continuing education for physiotherapists, occupational therapists, speech-language pathologists, and rehabilitation physicians. Other steps are utilising mobile rehabilitation units to reach patients in remote communities, implementing tele-rehabilitation services using secure, user-friendly digital platforms to extend reach and continuity of care and incorporating rehabilitation services into national trauma care guidelines and hospital discharge planning.

Countries like Australia and Canada have demonstrated the effectiveness of integrated rehabilitation services in trauma care, particularly through early intervention and community-based programs [17]. Malaysia could adopt similar strategies tailored to its health system structure and population needs. Evidence from a study showed that early, multidisciplinary rehabilitation can reduce hospital length of stay and readmission rates among trauma patients [23].

Investment in rehabilitation not only improves quality of life for survivors but also reduces long-term healthcare costs and societal burden due to disability. Collaboration with NGOs and international bodies, such as the International Society of Physical and Rehabilitation Medicine (ISPRM), could support capacity building and policy development in this area [24].

## Conclusions

Malaysia stands at a pivotal juncture in the evolution of its trauma care system. While the country has made significant strides in expanding universal health coverage, establishing specialised trauma centres, and training a competent trauma workforce, persistent gaps continue to undermine the equity, efficiency, and outcomes of trauma care. These include delays in emergency response, limited rural access, the absence of integrated trauma data systems, and inadequate rehabilitation infrastructure. To advance trauma care into a truly integrated system, Malaysia must adopt a multi-pronged, systems-level approach that addresses structural and operational deficiencies. Key imperatives include strengthening EMS coordination, implementing a national trauma registry, scaling rehabilitation services, and institutionalising trauma governance within the Ministry of Health. These reforms must be supported by cross-sectoral collaboration, robust data systems, and sustained political and financial commitment. Drawing on global best practices and regional experiences, Malaysia can position itself as a leader in trauma care reform within the ASEAN region. Such leadership will improve survival and recovery outcomes for trauma patients and contribute to the broader goals of health equity, resilience, and sustainable development. In the face of rising injury rates due to urbanisation, road congestion, and industrialisation, now is the time to act decisively. A comprehensive, inclusive, and data-informed trauma system is no longer aspirational; it is essential. With strategic investment and stakeholder engagement, Malaysia can build a trauma system that saves lives, restores function, and supports the long-term well-being of its citizens.
